# Mapping the evidence landscape of the oral microbiome-cancer relationship: insights from the 100 most-cited papers

**DOI:** 10.31744/einstein_journal/2026AO2412

**Published:** 2026-07-29

**Authors:** Lucas Calixto Amorim, Julia Nascimento Souza, Mel de Araújo Teixeira, Agnaldo Rocha de Souza, Victória Lopes Abdo, Priscila Bernadina Miranda Soares, Nidia Castro dos Santos, Aljomar José Vechiato-Filho, Jamil Awad Shibli, João Gabriel Silva Souza

**Affiliations:** 1 Faculdade de Ciências Odontológicas Montes Claros MG Brazil Faculdade de Ciências Odontológicas, Montes Claros, MG, Brazil.; 2 Hospital Israelita Albert Einstein Faculdade Israelita de Ciências da Saúde Albert Einstein São Paulo SP Brazil Faculdade Israelita de Ciências da Saúde Albert Einstein, Hospital Israelita Albert Einstein, São Paulo, SP, Brazil.; 3 Universidade Estadual de Montes Claros Faculdade de Odontologia Montes Claros MG Brazil Faculdade de Odontologia, Universidade Estadual de Montes Claros, Montes Claros, MG, Brazil.; 4 Universidade Guarulhos Guarulhos SP Brazil Dental Research Division, Universidade Guarulhos, Guarulhos, SP Brazil.; 5 Centro de Pesquisa do Câncer Oncovida Montes Claros MG Brazil Centro de Pesquisa do Câncer Oncovida, Montes Claros, MG, Brazil.; 6 ADA Forsyth Institute Somerville Massachusetts United States of America ADA Forsyth Institute, Somerville, Massachusetts, United States of America.; 7 Harvard School of Dental Medicine Department of Oral Medicine, Infection, and Immunity Boston Massachusetts United States of America Department of Oral Medicine, Infection, and Immunity, Harvard School of Dental Medicine, Boston, Massachusetts, United States of America.; 8 Department of Oral Health Sciences, Periodontology and Oral Microbiology Leuven KU Belgium Department of Oral Health Sciences, Periodontology and Oral Microbiology, KU Leuven, Belgium.; 9 Universidade Estadual de Campinas Faculdade de Odontologia de Piracicaba Piracicaba SP Brazil Faculdade de Odontologia de Piracicaba, Universidade Estadual de Campinas, Piracicaba, SP, Brazil.

**Keywords:** Neoplasms, Microbiota, Biofilms, Mouth

## Abstract

**Objective:**

This study aimed to qualitatively and quantitatively analyze the 100 most-cited papers investigating the relationship between the oral microbiome and cancer.

**Methods:**

A qualitative and quantitative bibliometric study was conducted to evaluate the 100 most-cited papers on the relationship between cancer and the oral microbiome. The search strategy was applied in the Scopus database. Data related to the articles, authors, and study characteristics were extracted. Descriptive and bivariate analyses were performed.

**Results:**

From the 1,033 records retrieved, the 100 most-cited papers in the field were ranked and selected. The most-cited article had accumulated 759 citations. The papers were published between 1998 and 2022; authors from North America and Asia were the most frequent contributors, and reviews were the most common study design. Oral, colorectal, esophageal, and pancreatic cancers were the most frequently investigated cancer sites. Funding was less frequently reported in review articles (OR=0.28; p=0.041) and in papers from Europe (OR=0.14; p<0.001), whereas funding support increased over time (OR=1.05; p=0.037).

**Conclusion:**

The most-cited literature indicates substantial research interest in the relationship between cancer and the oral microbiome. The most-cited papers show that this association has been investigated mainly in oral cancer, although associations with cancers at other sites have also been reported. Financial support and international collaboration may help broaden research on this topic.

## Highlights

Research interest in the relationship between the oral microbiome and cancer is increasing.

Oral cancer was the most frequently investigated cancer site in oral microbiome studies.

International collaboration may accelerate advances in this field.

Most highly cited papers were reviews, highlighting the need for more experimental studies.

## In Brief

This bibliometric study analyzed the 100 most-cited papers on cancer and the oral microbiome, highlighting publication trends, cancer sites, funding patterns, and international collaboration. The findings indicate growing interest in this topic and suggest that financial support and international collaboration may help expand research in this field.

## Graphical abstract

**Figure:**
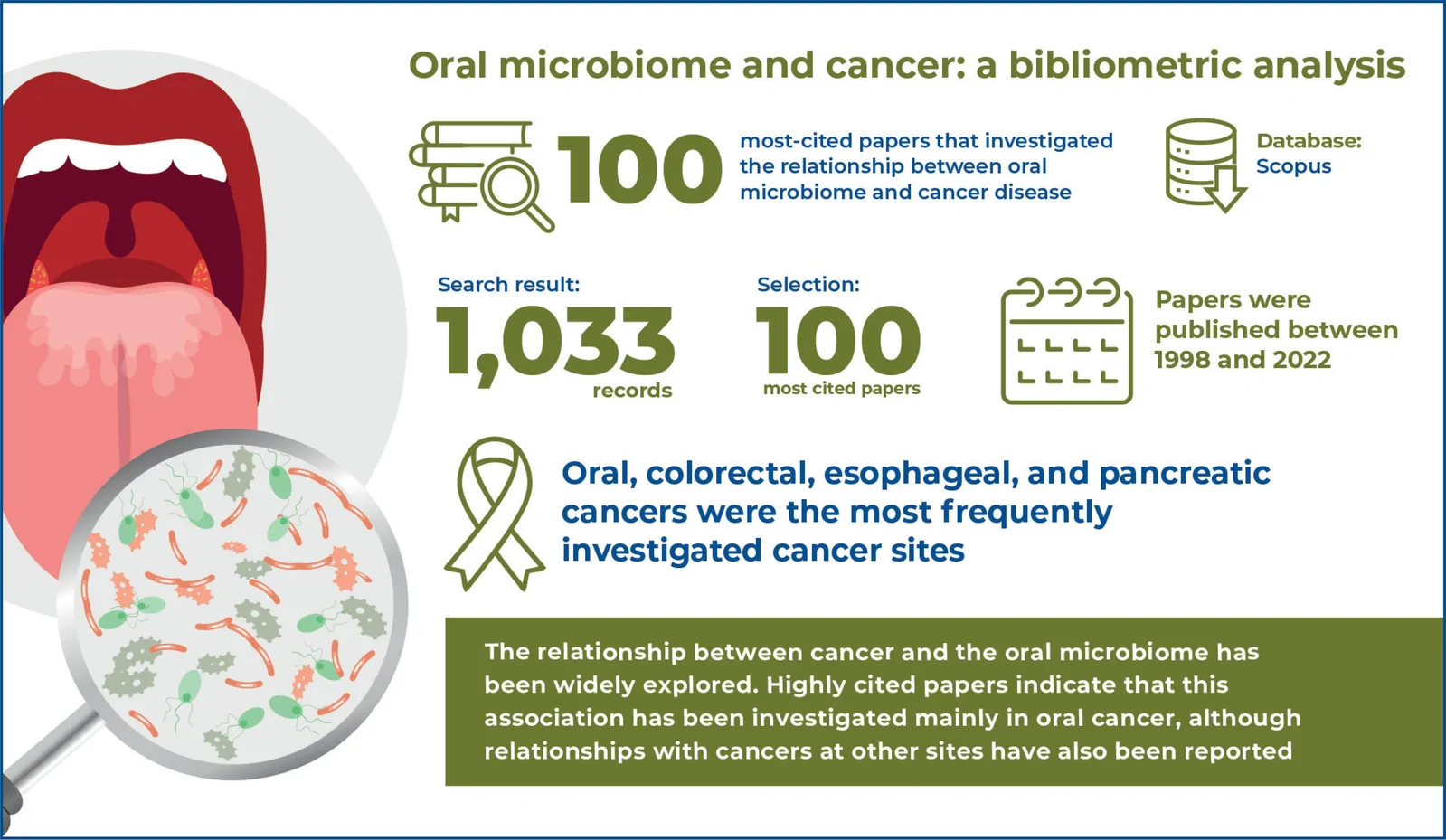

## INTRODUCTION

Cancer is the second leading cause of death worldwide and a major global health burden. Worldwide estimates suggest that one in five people will develop cancer during their lifetime, contributing to high mortality rates. According to GLOBOCAN statistics for 2022, lung, breast, and colorectal cancers are the most prevalent malignancies worldwide.^(1)^ Cancer also imposes a substantial burden on health systems because of the high costs associated with treatment, including hospitalization and palliative care.^(2)^ This disease has been associated with non-modifiable risk factors, such as genetic alterations, and modifiable risk factors, such as tobacco smoking, alcohol use, obesity, chronic inflammation, and infections.^(3)^ Beyond these well-recognized factors involved in the etiopathogenesis of malignant neoplasms, current evidence suggests that multiple biological mechanisms contribute to tumor development and progression.^(4)^

Among these mechanisms, evidence from in vitro and in vivo studies suggests that microorganisms may be involved in cancer progression.^(5)^ Bacterial cells may contribute to this process by modulating the host immune system and influencing tumor-related phenotypes.^(5)^ Within the human microbial ecosystem, the oral cavity harbors the second largest microbial community in the body, comprising more than 700 species, many of which have been associated with oral cancer and cancers at other body sites.^(6,7)^ Previous evidence has identified associations among alterations in the oral microbiome, increased colonization by microorganisms associated with periodontal diseases, such as Porphyromonas gingivalis,^(8)^ and long-term inflammatory responses that may contribute to cellular mutations and cancer cell proliferation.^(9–11)^ Patients with oral cancer have been shown to exhibit marked shifts in the salivary microbial profile compared with healthy individuals, suggesting a potential role for changes in the oral environment.^(12,13)^

Such microbiological changes have also been reported in patients with cancer at distant sites. Increased levels of periodontal pathogens have been detected in the subgingival biofilms of patients with gastric malignancies.^(14,15)^ Additionally, Fusobacterium nucleatum, a well-recognized oral pathogen, has been detected in colorectal cancer cells and metastatic tumors,^(16)^ and elevated levels of this species have been correlated with gastric tumor size.^(15)^ Because the oral microbiome is one of the main sources contributing to the pulmonary microbiome, evidence suggests that oral microorganisms are associated with inflammatory processes and lung cancer pathogenesis.^(17)^ Although some studies have focused on the relationship with the whole oral microbiome,^(7)^ defined as the entire microbial community residing in a specific habitat such as the oral cavity,^(6)^ other evidence has highlighted the role of specific components of the oral microbiome, such as Fusobacterium spp. and P. gingivalis.^(11,16)^

The relationship between cancer and the oral microbiome has therefore gained substantial scientific attention, as reflected in the rapid growth in related publications over the last decade. In this context, bibliometric studies can generate comprehensive data by systematically analyzing multiple indices, including citation counts for specific topics and broader scientific trends.^(18,19)^ Bibliometric studies have therefore been conducted across different fields to assess and characterize research activity within specific disciplines.^(20,21)^ Examining the most-cited papers in a field can provide valuable insights into influential publications, topic scope, and current research trends.^(18,19)^ However, no bibliometric analysis has specifically mapped the 100 most-cited papers on the relationship between cancer and the oral microbiome.

## OBJECTIVE

This study aimed to qualitatively and quantitatively analyze the 100 most-cited papers investigating the relationship between the oral microbiome and cancer.

## METHODS

### Design

A bibliometric study was conducted to qualitatively and quantitatively analyze the 100 most-cited papers on the relationship between cancer and the oral microbiome or individual oral microbial species.

### Search strategy

The search strategy combined terms related to the oral microbiome and cancer, including oral microbiome, oral microbiota, oral biofilm, Oral Microbiology, Salivary Microbiome, cancer, oral cancer, Squamous Cell Carcinoma, Neoplasms, and Carcinoma. The following search strategy was used: TITLE-ABS-KEY ("oral microbiome" OR "oral microbiota" OR "oral biofilm" OR "Oral Microbiology" OR "Salivary Microbiome") AND TITLE-ABS-KEY ("cancer" OR "oral cancer" OR "Squamous Cell Carcinoma" OR "Neoplasms" OR "Carcinoma"). Searches were conducted in the Scopus database in March 2025, with no restrictions on publication date or language. The search retrieved 1,033 records, which were ranked according to citation counts in Scopus. Study selection was performed independently by two examiners (L.C.A. and J.G.S.S.), with excellent inter-examiner agreement (κ>0.9). Titles and abstracts were screened to determine whether studies focused on the relationship between cancer and the oral microbiome. No restrictions were applied regarding cancer type or anatomical site, and studies evaluating any oral microbial species or the overall oral microbiome were considered eligible. When uncertainty remained after title and abstract screening, full-text articles were assessed to confirm eligibility. All study designs were considered. Disagreements between examiners were resolved by a third examiner. Citation counts reported in Scopus were considered, including total citation counts without excluding self-citations. After screening, the papers were ranked according to the total citation counts reported in Scopus. The 100 most-cited papers addressing the relationship between cancer and the oral microbiome were subsequently assessed in full text for data extraction.

### Data extraction

The following data were extracted from the selected papers: author names, article title, year of publication, journal, citation count, DOI, author affiliations, country and continent of the corresponding author, presence of international collaboration, study type/design, cancer site, oral microbial species or genera evaluated, funding information, and open-access status. International collaboration was defined as the presence of author affiliations from more than one country. Any funding source reported in the article was considered financial support.

### Statistics

GraphPad Prism (version 10.4.2 for macOS) was used to generate graphs, and SPSS (version 20.0) was used for statistical analyses. Descriptive analyses included absolute frequencies and proportions (%). Bivariate analyses were performed using the chi-square test, with odds ratios and 95% confidence intervals estimated. For the bivariate analyses, the dependent variables were funding support and international collaboration, which were analyzed separately. The independent variables evaluated for association with each dependent variable included cancer site, open-access status, study design, continent, year of publication, and citation count. A significance level of 5% was adopted.

## RESULTS

From the 1,033 records retrieved, the 100 most-cited papers in the field were ranked and selected. The most-cited article had accumulated 759 citations in Scopus over a 6-year period, from its publication in 2019 to the search date in 2025, whereas the paper ranked 100th had received 52 citations (Table 1). All included articles were published between 1998 and 2022, with a publication peak in 2019, which accounted for 19 papers (Figure 1A). North America and Asia contributed the largest number of highly cited papers, with 38 and 37 publications, respectively; however, most papers did not involve international collaboration (Figure 1B). Regarding study design, reviews were the most prevalent (n=40), followed by cross-sectional observational studies (n=28) (Figure 1C). The United States of America was the country with the highest number of publications among the 100 most-cited papers (Figure 1D).

**Table 1:** Top 100 most-cited papers on the relationship between the oral microbiome and cancer according to Scopus, ranked by citation count

Rank	Reference	Year	Citations
1	Brennan C.A.; et al. Fusobacterium nucleatum — symbiont, opportunist and oncobacterium. Nature Reviews Microbiology 2019;17(3):156-166.	2019	759
2	Fan X.; et al. Human oral microbiome and prospective risk for pancreatic cancer: A population-based nested case-control study. Gut. 2018;67(1):120-127.	2018	576
3	Abed J.; et al. Fap2 Mediates Fusobacterium nucleatum Colorectal Adenocarcinoma Enrichment by Binding to Tumor-Expressed Gal-GalNAc. Cell Host and Microbe. 2016;20(2):215-25.	2016	570
4	Farrell J.J.; et al. Variations of oral microbiota are associated with pancreatic diseases, including pancreatic cancer. Gut. 2012;61(4):582-8.	2012	519
5	Flemer B.; et al. The oral microbiota in colorectal cancer is distinctive and predictive Gut. 2018;67(8):1454-1463.	2018	447
6	Zhao H.; et al. Variations in oral microbiota associated with oral cancer. Scientific Reports. 2017;7(1):11773.	2017	307
7	Peters B.A.; et al. Oral microbiome composition reflects prospective risk for esophageal cancers. Cancer Research 2017;77(23):6777-6787.	2017	297
8	Schmidt B.L.; et al. Changes in the abundance of oral microbiota associated with oral cancer. PLoS ONE. 2014;9(6):e98741.	2014	296
9	Chattopadhyay I.; et al. Role of Oral Microbiome Signatures in Diagnosis and Prognosis of Oral Cancer. Technology in Cancer Research and Treatment. 2019;18:1533033819867354.	2019	275
10	Warren R.L.; et al. Co-occurrence of anaerobic bacteria in colorectal carcinomas. Microbiome 2013;1(1):16.	2013	266
11	Nagy K.N.; et al. The microflora associated with human oral carcinomas Oral Oncology. 1998;34(4):304-8.	1998	263
12	Yang C.-Y.; et al. Oral microbiota community dynamics associated with oral squamous cell carcinoma staging. Frontiers in Microbiology 2018;9:862.	2018	257
13	Zhang L.; et al. The Oral Microbiota May Have an Influence on Oral Cancer. Frontiers in Cellular and Infection Microbiology. 2020;9:476.	2020	252
14	Karpiński T.M.; et al. Role of oral microbiota in cancer development. Microorganisms 2019;7(1):20.	2019	252
15	Pushalkar S.; et al. Comparison of oral microbiota in tumor and non-tumor tissues of patients with oral squamous cell carcinoma. BMC Microbiology. 2012;12:144.	2012	252
16	Radaic A.; et al. The oralome and its dysbiosis: New insights into oral microbiome-host interactions Computational and Structural Biotechnology Journal. 2021;19:1335-1360.	2021	245
17	Hayes R.B.; et al. Association of oral microbiome with risk for incident head and neck squamous cell cancer. JAMA Oncology 2018;4(3):358-365.	2018	244
18	Drewes J.L.; et al. High-resolution bacterial 16S rRNA gene profile meta-analysis and biofilm status reveal common colorectal cancer consortia. npj Biofilms and Microbiomes 2017;3:34.	2017	237
19	Ahn J.; et al. Oral microbiome and oral and gastrointestinal cancer risk. Cancer Causes and Control. 2012;23(3):399-404.	2012	194
20	Gholizadeh P.; et al. Role of oral microbiome on oral cancers, a review. Biomedicine and Pharmacotherapy. 2016;84:552-558.	2016	193
21	Kapila Y.L.; et al. Oral health's inextricable connection to systemic health: Special populations bring to bear multimodal relationships and factors connecting periodontal disease to systemic diseases and conditions. Periodontology 2000 2021;87(1):11-16.	2021	190
22	Irfan M.; et al. The Oral Microbiome and Cancer Frontiers in Immunology. 2020;11:591088.	2020	190
23	Chen X.; et al. Oral microbiota and risk for esophageal squamous cell carcinoma in a high-risk area of China. PLoS ONE. 2015;10(12):e0143603.	2015	180
24	Hong B.-Y.; et al. Chemotherapy-induced oral mucositis is associated with detrimental bacterial dysbiosis Microbiome. 2019;7(1):66.	2019	177
25	Koliarakis I.; et al. Oral bacteria and intestinal dysbiosis in colorectal cancer. International Journal of Molecular Sciences. 2019;20(17):4146.	2019	176
26	Lee W.-H.; et al. Bacterial alterations in salivary microbiota and their association in oral cancer. Scientific Reports. 2017;7(1):16540.	2017	161
27	Gaiser R.A.; et al. Enrichment of oral microbiota in early cystic precursors to invasive pancreatic cancer. Gut 2019;68(12):2186-2194.	2019	161
28	Torres P.J.; et al. Characterization of the salivary microbiome in patients with pancreatic cancer. PeerJ 2015;3:e1373.	2015	158
29	Lu M.; et al. Oral microbiota: A new view of body health. Food Science and Human Wellness 2019;8(1):8-15.	2019	141
30	Porter C.M.; et al. The microbiome in prostate inflammation and prostate cancer Prostate Cancer and Prostatic Diseases. 2018;21(3):345-354.	2018	141
31	Meurman J.H.; et al. Oral microbiota and cancer. Journal of Oral Microbiology 2010;2.	2010	137
32	Tuominen H.; et al. Oral Microbiota and Cancer Development. Pathobiology. 2021;88(2):116-126.	2021	133
33	Ganly I.; et al. Periodontal pathogens are a risk factor of oral cavity squamous cell carcinoma, independent of tobacco and alcohol and human papillomavirus. International Journal of Cancer. 2019;145(3):775-784.	2019	130
34	Bertolini M.; et al. Candida albicans induces mucosal bacterial dysbiosis that promotes invasive infection PLoS Pathogens. 2019;15(4):e1007717.	2019	128
35	Stasiewicz M.; et al. The oral microbiota and its role in carcinogenesis. Seminars in Cancer Biology. 2022;86(Pt 3):633-642.	2022	127
36	Flynn K.J.; et al. Metabolic and community synergy of oral bacteria in colorectal cancer. mSphere 2016;1(3):e00102-16.	2016	124
37	Zhu X.-X.; et al. The Potential Effect of Oral Microbiota in the Prediction of Mucositis During Radiotherapy for Nasopharyngeal Carcinoma EBioMedicine. 2017;18:23-31.	2017	120
38	Hernandez B.Y.; et al. Betel nut chewing, oral premalignant lesions, and the oral microbiome PLoS ONE. 2017;12(2):e0172196.	2017	120
39	Hsiao J.-R.; et al. The interplay between oral microbiome, lifestyle factors and genetic polymorphisms in the risk of oral squamous cell carcinoma Carcinogenesis. 2018;39(6):778-787.	2018	114
40	Kavarthapu A.; et al. Linking chronic periodontitis and oral cancer: A review. Oral Oncology. 2021;121:105375.	2021	112
41	Lim Y.; et al. Oral microbiome: A new biomarker reservoir for oral and oropharyngeal cancers. Theranostics. 2017;7(17):4313-4321.	2017	106
42	Singh H.; et al. Gastrointestinal and oral microbiome signatures associated with healthy aging. GeroScience. 2019;41(6):907-921.	2019	105
43	Di Cosola M.; et al. Candida albicans and oral carcinogenesis. A brief review Journal of Fungi. 2021;7(6):476.	2021	103
44	Yost S.; et al. Increased virulence of the oral microbiome in oral squamous cell carcinoma revealed by metatranscriptome analyses International Journal of Oral Science. 2018;10(4):32.	2018	103
45	La Rosa G.R.M.; et al. Association of oral dysbiosis with oral cancer development (Review) Oncology Letters. 2020;19(4):3045-3058.	2020	101
46	Yang S.-F.; et al. Compositional and functional variations of oral microbiota associated with the mutational changes in oral cancer. Oral Oncology 2018;77:1-8.	2018	101
47	Börnigen D.; et al. Alterations in oral bacterial communities are associated with risk factors for oral and oropharyngeal cancer. Scientific Reports. 2017;7(1):17686.	2017	100
48	Fábian T.K.; et al. Salivary genomics, transcriptomics, and proteomics: The emerging concept of the oral ecosystem and their use in the early diagnosis of cancer and other diseases. Current Genomics. 2008;9(1):11-21.	2008	100
49	Zheng D.-W.; et al. Biomaterial-mediated modulation of oral microbiota synergizes with PD-1 blockade in mice with oral squamous cell carcinoma Nature Biomedical Engineering. 2022;6(1):32-43.	2022	99
50	Atanasova K.R.; et al. Looking in the Porphyromonas gingivalis cabinet of curiosities: The microbium, the host, and cancer association. Molecular Oral Microbiology. 2014;29(2):55-66.	2014	97
51	Sun J.; et al. Role of the oral microbiota in cancer evolution and progression. Cancer Medicine. 2020;9(17):6306-6321.	2020	97
52	Su S.-C.; et al. Oral microbial dysbiosis and its performance in predicting oral cancer. Carcinogenesis. 2021;42(1):127-135.	2021	95
53	Wang H.; et al. Microbiomic differences in tumor and paired-normal tissue in head and neck squamous cell carcinomas Genome Medicine. 2017;9(1):14.	2017	94
54	Kato I.; et al. Nutritional Correlates of Human Oral Microbiome. Journal of the American College of Nutrition. 2017;36(2):88-98.	2017	94
55	Michaud D.S.; et al. Microbiota, oral microbiome, and pancreatic cancer. Cancer Journal (United States) 2014;20(3):203-6.	2014	94
56	Lim Y.; et al. The performance of an oral microbiome biomarker panel in predicting oral cavity and oropharyngeal cancers Frontiers in Cellular and Infection Microbiology. 2018;8:267.	2018	91
57	Russo E.; et al. Preliminary comparison of oral and intestinal human microbiota in patients with colorectal cancer: A pilot study. Frontiers in Microbiology. 2018;8:2699.	2018	90
58	Al-Hebshi N.N.; et al. Robust species taxonomy assignment algorithm for 16S rRNA NGS reads: Application to oral carcinoma samples Journal of Oral Microbiology. 2015;7:28934.	2015	90
59	Yang Y.; et al. Prospective study of oral microbiome and colorectal cancer risk in low-income and African American populations International Journal of Cancer. 2019;144(10):2381-2389.	2019	89
60	Hou J.; et al. Distinct shifts in the oral microbiota are associated with the progression and aggravation of mucositis during radiotherapy Radiotherapy and Oncology. 2018;129(1):44-51.	2018	86
61	Rai A.K.; et al. Dysbiosis of salivary microbiome and cytokines influence oral squamous cell carcinoma through inflammation. Archives of Microbiology. 2021;203(1):137-152.	2021	85
62	Lafuente Ibáñez de Mendoza I.; et al. Role of Porphyromonas gingivalis in oral squamous cell carcinoma development: A systematic review Journal of Periodontal Research. 2020;55(1):13-22.	2020	82
63	Yang J.; et al. Dysbiosis of the Salivary Microbiome Is Associated With Non-smoking Female Lung Cancer and Correlated With Immunocytochemistry Markers Frontiers in Oncology. 2018;8:520.	2018	82
64	Meurman J.H.; et al. Oral micro-organisms in the etiology of cancer Acta Odontologica Scandinavica. 2008;66(6):321-6.	2008	80
65	Kawasaki M.; et al. Oral infectious bacteria in dental plaque and saliva as risk factors in patients with esophageal cancer. Cancer. 2021;127(4):512-519.	2021	80
66	Healy C.M.; et al. The microbiome and oral cancer: More questions than answers Oral Oncology. 2019;89:30-33.	2019	78
67	Wang L.; et al. The oral microbiome and oral cancer. Clinics in Laboratory Medicine 2014;34(4):711-9.	2014	78
68	Maddi A.; et al. The microbiome and lung cancer. Journal of Thoracic Disease 2019;11(1):280-291.	2019	76
69	Le Bars P.; et al. The oral cavity microbiota: Between health, oral disease, and cancers of the aerodigestive tract Canadian Journal of Microbiology. 2017;63(6):475-492.	2017	76
70	Mascitti M.; et al. Beyond head and neck cancer: The relationship between oral microbiota and tumour development in distant organs. Frontiers in Cellular and Infection Microbiology. 2019;9:232.	2019	73
71	Cueva C.; et al. Interplay between dietary polyphenols and oral and gut microbiota in the development of colorectal cancer. Nutrients. 2020;12(3):625.	2020	72
72	Kageyama S.; et al. Characteristics of the salivary microbiota in patients with various digestive tract cancers Frontiers in Microbiology. 2019;10:1780.	2019	71
73	Olson S.H.; et al. The oral microbiota in patients with pancreatic cancer, patients with IPMNs, and controls: a pilot study Cancer Causes and Control. 2017;28(9):959-969.	2017	71
74	Ramage G.; et al. Antifungal, cytotoxic, and immunomodulatory properties of tea tree oil and its derivative components: Potential role in management of oral candidosis in cancer patients Frontiers in Microbiology. 2012;3:220.	2012	71
75	Sun J.-H.; et al. A screening method for gastric cancer by oral microbiome detection Oncology Reports. 2018;39(5):2217-2224.	2018	71
76	Zhang W.; et al. Salivary microbial dysbiosis is associated with systemic inflammatory markers and predicted oral metabolites in non-small cell lung cancer patients. Journal of Cancer. 2019;10(7):1651-1662.	2019	70
77	Zhang S.; et al. Human oral microbiome dysbiosis as a novel non-invasive biomarker in detection of colorectal cancer. Theranostics. 2020;10(25):11595-11606.	2020	69
78	Galvão-Moreira L.V.; et al. Oral microbiome, periodontitis and risk of head and neck cancer. Oral Oncology. 2016;53:17-9.	2016	69
79	Gnanasekaran J.; et al. Intracellular Porphyromonas gingivalis promotes the tumorigenic behavior of pancreatic carcinoma cells. Cancers. 2020;12(8):2331.	2020	69
80	Wolf A.; et al. The salivary microbiome as an indicator of carcinogenesis in patients with oropharyngeal squamous cell carcinoma: A pilot study Scientific Reports. 2017;7(1):5867.	2017	68
81	Hu J.; et al. Variations of Tongue Coating Microbiota in Patients with Gastric Cancer. BioMed Research International. 2015;2015:173729.	2015	68
82	Hosgood H.D.; et al. Variation in oral microbiome is associated with future risk of lung cancer among never-smokers Thorax. 2021;76(3):256-263.	2021	67
83	Hu Y.-J.; et al. Exploring the Dynamic Core Microbiome of Plaque Microbiota during Head-and-Neck Radiotherapy Using Pyrosequencing. PLoS ONE. 2013;8(2):e56343.	2013	66
84	Sarkar P.; et al. Dysbiosis of Oral Microbiota During Oral Squamous Cell Carcinoma Development Frontiers in Oncology. 2021;11:614448.	2021	65
85	Sami A.; et al. The role of the microbiome in oral squamous cell carcinoma with insight into the microbiome–treatment axis International Journal of Molecular Sciences. 2020;21(21):8061.	2020	65
86	Chen M.-F.; et al. Porphyromonas gingivalis promotes tumor progression in esophageal squamous cell carcinoma Cellular Oncology. 2021;44(2):373-384.	2021	63
87	Mohammed H.; et al. Oral dysbiosis in pancreatic cancer and liver cirrhosis: A review of the literature. Biomedicines. 2018;6(4):115.	2018	62
88	Al-Hebshi N.N.; et al. The Microbiome of Oral Squamous Cell Carcinomas: a Functional Perspective Current Oral Health Reports. 2019;6:145-160.	2019	62
89	Dong J.; et al. Relationships Between Oral Microecosystem and Respiratory Diseases Frontiers in Molecular Biosciences. 2022;8:718222.	2022	61
90	Vesty A.; et al. Oral microbial influences on oral mucositis during radiotherapy treatment of head and neck cancer. Supportive Care in Cancer. 2020;28(6):2683-2691.	2020	61
91	Freudenheim J.L.; et al. Periodontal disease and breast cancer: Prospective cohort study of postmenopausal women Cancer Epidemiology, Biomarkers and Prevention. 2016;25(1):43-50.	2016	60
92	Wang Q.; et al. Oral Microbiome in Patients with Oesophageal Squamous Cell Carcinoma Scientific Reports. 2019;9(1):19055.	2019	59
93	Neuzillet C.; et al. Prognostic value of intratumoral Fusobacterium nucleatum and association with immune-related gene expression in oral squamous cell carcinoma patients Scientific Reports. 2021;11(1):7870.	2021	58
94	Marttila E.; et al. Acetaldehyde production and microbial colonization in oral squamous cell carcinoma and oral lichenoid disease. Oral Surgery, Oral Medicine, Oral Pathology and Oral Radiology 2013;116(1):61-8.	2013	58
95	Teles F.R.F.; et al. Association or Causation? Exploring the Oral Microbiome and Cancer Links Journal of Dental Research. 2020;99(13):1411-1424.	2020	57
96	Wei A.L.; et al. Oral microbiome and pancreatic cancer. World Journal of Gastroenterology. 2020;26(48):7679-7692.	2020	56
97	Stashenko P.; et al. The oral mouse microbiome promotes tumorigenesis in oral squamous cell carcinoma. mSystems 2019;4(4):e00323-19.	2019	55
98	Vesty A.; et al. Microbial and inflammatory-based salivary biomarkers of head and neck squamous cell carcinoma Clinical and Experimental Dental Research. 2018;4(6):255-262.	2018	53
99	Park S.Y.; et al. Oral–gut microbiome axis in gastrointestinal disease and cancer Cancers. 2021 Apr 28;13(9):2124.	2021	53
100	Wei X.; et al. Molecular profiling of oral microbiota in jawbone samples of bisphosphonate-related osteonecrosis of the jaw Oral Diseases. 2012;18(6):602-12.	2012	52

Citations reflect Scopus citation counts retrieved at the time of the search (2025).

**Figure 1:**
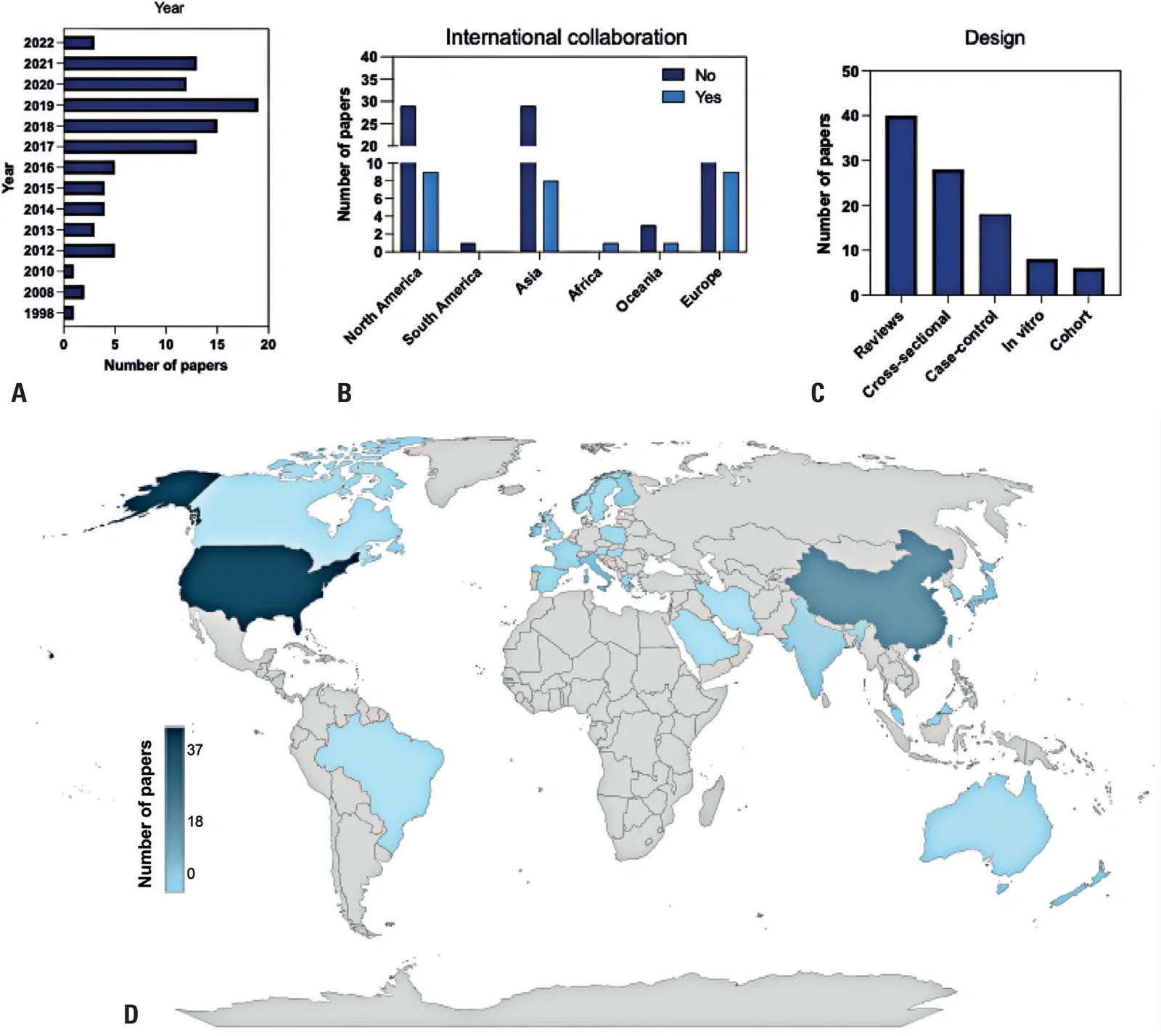
Scientific patterns of the top 100 most-cited papers on the relationship between cancer and the oral microbiome. A) Number of papers by year of publication. B) Presence or absence of international collaboration by continent of the corresponding author. International collaboration was defined as the inclusion of authors from more than one country. C) Study design/type. D) Geographic heat map showing the number of papers per country. Countries were assigned based on the affiliation of the corresponding author

Oral, colorectal, esophageal, and pancreatic cancers were the most frequently investigated cancer sites among the highly cited papers (Figure 2A). Regarding microbial targets, most studies focused on the overall oral microbiome without restricting the analysis to specific species or genera (Figure 2B). Nevertheless, several bacterial species or genera were repeatedly examined, particularly Streptococcus spp., Porphyromonas gingivalis, and Fusobacterium nucleatum. When funding was evaluated according to study design, most case–control and in vitro studies reported financial support (Figure 2C). With respect to citation performance, papers with reported funding showed higher mean citation counts descriptively (Figure 2D). The two continents that contributed the largest number of highly cited papers, North America and Asia, also showed higher proportions of funded studies (Figure 2E). No clear pattern was observed between cancer site and funding status; however, most studies focusing on oral and colorectal cancers reported funding support (Figure 2F). Papers addressing colorectal, pancreatic, and oral cancers also tended to show higher citation counts (Figure 2G). Scientific Reports and Oral Oncology had the highest number of publications among the 100 most-cited papers on the relationship between cancer and the oral microbiome, with 6 and 5 papers, respectively (Table 2).

**Figure 2:**
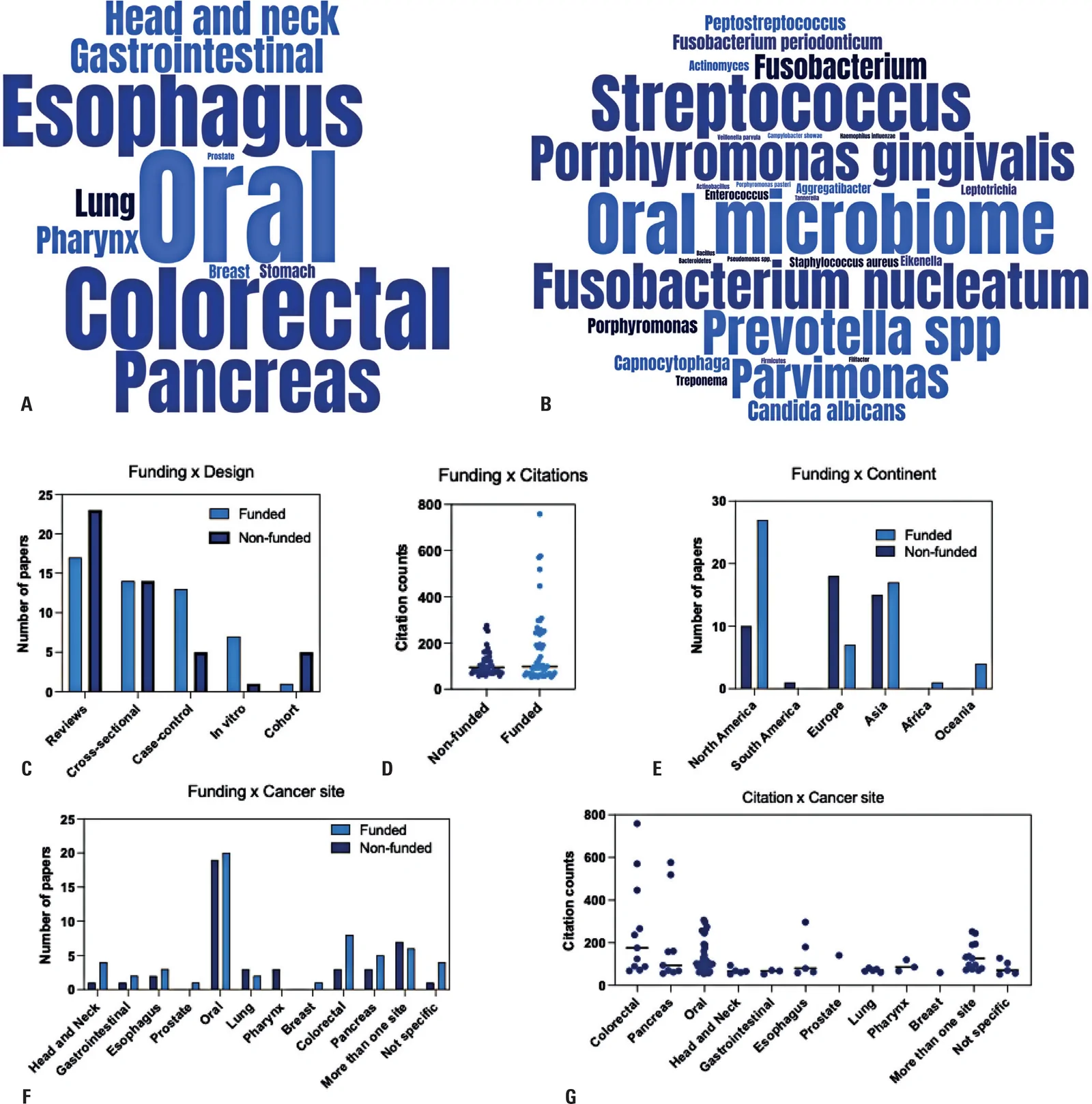
Scientific profile of the 100 most-cited papers on the relationship between cancer and the oral microbiome. A) Word cloud of the cancer types/sites evaluated. B) Word cloud of the bacterial species/genera evaluated. C) Funding reported by study design. D) Number of papers and mean citation counts according to financial support. E) Funding reported by the continent of the corresponding author. F) Funding status (reported vs. not reported) by cancer site evaluated. G) Citation counts by cancer site evaluated

**Table 2:** Top five journals publishing the most-cited papers on the relationship between cancer and the oral microbiome, ranked by number of papers

Journal	Number of papers	Citations
Scientific Reports	6	753
Oral Oncology	5	623
Frontiers in Microbiology	4	489
Gut	4	1,703
PLoS ONE	4	662

Note: Citations represent total Scopus citation counts for the papers included in this analysis.

In the bivariate analysis evaluating associations between funding support and other study characteristics among the 100 most-cited papers, funding was less frequently reported in review papers than in case–control studies (OR=0.28; p=0.041) and in papers from Europe than in those from North America (OR=0.14; p<0.001). In contrast, the likelihood of funding support increased over the years (OR=1.05; p=0.037) (Table 3). No variable showed a statistically significant (p<0.05) relationship with international collaboration (Table 3). However, studies with international collaboration showed higher odds of being published in an open-access format, although this association did not reach statistical significance (p=0.098).

**Table 3:** Bivariate analysis of factors associated with funding support and international collaboration using the chi-square test

Variable		Study funded		With international collaboration	
		OR (95% CI)	p-value	OR (95% CI)	p value
Funding					
	No			1	
	Yes			1.02 (0.40-2.62)	0.954
International collaboration					
	No	1			
	Yes	1.02 (0.40-2.62)	0.954		
Cancer site					
	More than one site	1		1	
	Gastrointestinal	2.33 (0.16-32.58)	0.529	2.75 (0.16-46.79)	0.484
	Esophagus	1.75 (0.21-14.22)	0.601	1.37 (0.09-19.64)	0.814
	Lung	-	-	2.75 (0.16-46.79)	0.484
	Colorectal	3.11 (0.55-17.33)	0.195	1.22 (0.14-10.48)	0.855
	Pancreas	1.45 (0.26-8.04)	0.665	4.40 (0.59-32.50)	0.146
	Oral	1.22 (0.34-4.32)	0.749	2.16 (0.41-11.36)	0.363
	Head and neck	4.66 (0.40-53.95)	0.217	-	-
	Others/not specific	2.33 (0.46-11.80)	0.306	0.50 (0.03-6.35)	0.593
Open access					
	No	1		1	
	Yes	2.77 (0.93-8.24)	0.066	5.77 (0.72-46.10)	0.098
Study design					
	Case–control	1		1	
	Cross-sectional	0.38 (0.10-1.36)	0.140	0.66 (0.18-2.44)	0.541
	Review	0.28 (0.08-0.95)	0.041	0.35 (0.09-1.30)	0.119
	Cohort	1.92 (0.17-20.81)	0.591	0.40 (0.03-4.23)	0.447
	In vitro	2.69 (0.26-27.82)	0.406	1.20 (0.21-6.80)	0.837
Continent					
	North America	1		1	
	South America/Africa/Oceania	1.85 (0.19-17.85)	0.594	1.81 (0.28-11.75)	0.533
	Europe	0.14 (0.04-0.44)	<0.001	1.41 (0.43-4.55)	0.566
	Asia	0.42 (0.15-1.14)	0.090	0.83 (0.25-2.73)	0.768
	Year	1.05 (1.00-1.01)	0.037	1.00 (0.99-1.00)	0.359
	Citation counts	1.04 (0.93-1.16)	0.465	0.91 (0.81-1.03)	0.168

OR: odds ratio; 95% CI: 95% confidence interval.

Reference categories: Study funded — No; International Collaboration — No; Cancer site — More than one site; Open Access — No; Study design — Case–control; Continent — North America. Dashes (–) indicate that the model could not be estimated for this category.

## DISCUSSION

Oral microorganisms contribute to prevalent oral conditions and have been associated with the progression of various systemic diseases, including cancer.^(22–24)^ Although these organisms have not been shown to cause cancer, their presence has been associated with modulation of the host immune system, the release of bioactive metabolites, and inflammatory processes involved in tumor progression.^(5)^ Although some mechanisms and roles of oral microorganisms in cancer, particularly at sites distant from the oral cavity, remain unclear, mapping trends among the most-cited papers may help identify research patterns and underexplored areas in this field. Here, we identified the most-cited papers published up to 2025 that evaluated the relationship between cancer and the oral microbiome. The findings showed that most papers were published within the last seven years before the search date, including the most-cited paper, which was published in 2019. Most papers were reviews and were published mainly by authors from North America and Asia; funding support was less frequently reported when corresponding authors were from South America, Africa, or Oceania. Although most papers focused on the relationship between oral bacteria and oral cancer, a trend was also observed toward studies evaluating specific oral bacterial species and their roles in cancers at other human sites, such as colorectal, esophageal, and pancreatic cancers.

In vivo evidence has demonstrated marked changes in the salivary and local microbiota of patients with oral cancer, suggesting a potential role of the microbiome in inflammatory processes and immune responses.^(25)^ Because oral microorganisms are closely associated with oral surfaces and oral biofilms have a well-recognized role in oral inflammatory diseases, the association between the oral microbiome and cancer has gained increasing attention, which may explain the growing number of published papers. The translocation of oral microorganisms to distant human sites is also well recognized, and these organisms have been associated with various systemic conditions, such as diabetes, Alzheimer's disease, and cardiovascular disease.^(26,27)^ Accordingly, researchers have increasingly evaluated the role of the oral microbiome in cancers at sites distant from the oral cavity. Among the most-cited papers, studies investigated the relationship between the oral microbiome and cancers of the esophagus, colorectum, pancreas, and other sites. Because this analysis focused on the most-cited papers, more recent studies not included in the top 100 may have explored these associations using newer experimental approaches and may provide further insights into the field.

High-throughput techniques have enabled researchers to gain a more detailed understanding of the oral microbiome and the microbial communities that colonize oral surfaces. Although most included studies focused on the effects of the overall oral microbiome on cancer, some examined specific bacterial species or genera, particularly abundant genera such as Streptococcus and well-recognized oral pathogens such as *Porphyromonas gingivalis* and *Fusobacterium nucleatum*. *F. nucleatum* has been detected in metastatic cells associated with colorectal cancer,^(16)^ and *P. gingivalis* has been implicated in inducing an inflammatory microenvironment in orodigestive carcinomas.^(28)^ These findings suggest that research in this field has increasingly focused on specific microbial targets, rather than solely evaluating the potential role of the overall oral microbiome, to better characterize individual organisms, their mechanisms, and their effects. Thus, highly cited papers focusing on specific questions and microbial targets appear to have been well recognized in the field.

Although evidence regarding the role of oral microorganisms in cancer has received considerable attention over the last decade, most highly cited papers in this analysis were reviews. Meta-trend studies have shown that review articles are more likely to receive higher citation counts, with estimates indicating that reviews can be cited up to three times more often than original research articles.^(29)^ However, nonsystematic narrative reviews are particularly prone to citation bias and may fail to adequately acknowledge the original sources of information.^(30)^ Therefore, although review papers elucidating the role of oral microorganisms in cancer have been widely cited, further original research may be needed to generate new evidence in the field.

Funding is essential for advancing scientific knowledge and supporting health research. Although journals require the declaration of financial support, some do not provide standardized submission protocols, which may lead to biased reporting and underestimation of funding.^(31)^ Among the 100 most-cited papers, funding was more frequently reported in highly cited articles, studies focusing on oral cancer, case–control studies, and papers published by corresponding authors from North America. The bivariate analysis showed a significantly lower likelihood of funding being reported in Europe than in North America. Moreover, review papers were less likely to report funding. The profile identified may help authors and stakeholders understand funding patterns in this field and identify areas that may benefit from further support.

The limitations of the present study include the use of a single database, as other databases may contain different studies and citation counts, potentially influencing the findings. Therefore, including other databases may affect the ranking and profile presented. Although this approach has been used in previous bibliometric studies,^(18,21)^ incorporating additional databases may highlight different aspects of the topic. Moreover, focusing on the most-cited papers limits the inclusion of more recently published studies. However, highly cited papers are generally considered influential contributions in the field and may help researchers identify publication patterns in this area.

## CONCLUSION

This bibliometric analysis shows that research on the relationship between cancer and the oral microbiome has expanded substantially over the past decade, with a strong emphasis on oral cancer and review-based publications. Highly cited papers predominantly originated from North America and Asia and focused on both the overall oral microbiome and specific bacterial taxa, whereas funding support increased over time but remained unevenly distributed across regions and study designs. These findings suggest that the mapped literature may have gaps regarding mechanistic and experimental studies on cancers at sites distant from the oral cavity and highlight the importance of sustained funding and international collaboration in supporting further research on the oral microbiome–cancer relationship.

## Data Availability

The underlying content is contained within the manuscript.
